# YOT-Net: YOLOv3 Combined Triplet Loss Network for Copper Elbow Surface Defect Detection

**DOI:** 10.3390/s21217260

**Published:** 2021-10-31

**Authors:** Yuanqing Xian, Guangjun Liu, Jinfu Fan, Yang Yu, Zhongjie Wang

**Affiliations:** 1 College of Electronics and Information Engineering, Tongji University, Shanghai 201804, China; xianyuanqing@tongji.edu.cn (Y.X.); fanjinfu@tongji.edu.cn (J.F.); 1510471@tongji.edu.cn (Y.Y.); 2 School of Mathematics and Computer Science, Guangdong Ocean University, Zhanjiang 524088, China; 3 School of Mechanical Engineering, Tongji University, Shanghai 201804, China; gjliu@tongji.edu.cn

**Keywords:** copper elbow, defect detection, YOLOv3, triplet loss, deep learning

## Abstract

Copper elbows are an important product in industry. They are used to connect pipes for transferring gas, oil, and liquids. Defective copper elbows can lead to serious industrial accidents. In this paper, a novel model named YOT-Net (YOLOv3 combined triplet loss network) is proposed to automatically detect defective copper elbows. To increase the defect detection accuracy, triplet loss function is employed in YOT-Net. The triplet loss function is introduced into the loss module of YOT-Net, which utilizes image similarity to enhance feature extraction ability. The proposed method of YOT-Net shows outstanding performance in copper elbow surface defect detection.

## 1. Introduction

Quality control is a key problem in industry. Surface defect detection plays an important role in quality control tasks. Many industry companies pay much attention to developing surface detection technology, and spend much effort and money on this endeavour.

In practice, the task of surface defect detection is mostly conducted manually by workers. Since worker energy is limited and the task is boring, after a long, continuous work-time, defect detection decreases. To deal with this problem, it is necessary to perform surface defect detection tasks automatically so as to enhance detection efficiency.

With the rapid development of AI technology, automatic surface defect detection has become a promising area in recent years. The current methods can be mainly divided into two categories. One is based on traditional methods, while the other is based on machine learning methods.

### 1.1. Current Methods

#### 1.1.1. The Traditional Detection Approaches

Most early studies on surface defect detection use artificial design features to achieve good results [1,2,3]. These methods heavily depend on professional domain knowledge, and are subject to specific conditions.

The traditional approach uses hand-crafted features to detect defects. There are four kinds of approaches, i.e, spectral-based, statistical-based, threshold-based, and model-based methods [4]. For example, the spectral-based method used in defect detection tasks involves Fourier transform [5], Gabor filters [6], and Wavelet transform [7,8].

Although the traditional methods have achieved good performance in surface defect detection, they are very sensitive to illumination and background. The use of traditional image processing methods to solve the problem of surface defects relies on images with uniform illumination and obvious surface defects. However, in the actual complex industrial environment, there are situations such as small differences between defect imaging and background, low contrast, large variation of defect scale and various types, or a large amount of noise in the defect image. These issues make the methods difficult to deploy. To solve these problems, machine learning methods are employed to detect surface defects.

#### 1.1.2. Detection Approaches Based on Machine Learning

A. Shallow Learning

Shallow learning was a popular method to detect surface defects in the past decades. These methods mainly include Bayesian algorithm [9], KNN [10], NNs [11], SVM [12], and so on. Ref. [13] utilizes Bayesian network to classify steel surfaces. Ref. [14] uses k-nearest neighbor algorithm to inspect rail surfaces. Ref. [15] utilizes a small set of wavelet features and employs support vector machine methods for defect detection. However, shallow learning lacks the ability to represent the complex function and make it restricitive of generalization ability for hard classification problems. Otherwise, shallow learning methods rely more on human expertise, i.e., SVM suffers from artificial feature extraction, KNN depends on choosing a good category number to classify.

Shallow learning methods still cannot achieve satisfied detection accuracy although they are more universal than the traditional methods. These methods, which can be called shallow methods compared with the deep learning method, are more stable and less dependent on human knowledge. These shallow methods achieve appreciated results in the defect detection domain. Now that the computation power has greatly improved, large neural networks have been introduced into the defect detection problem and the results, so far, have proved to be satisfactory.

B. Deep Learning

As the big data era began, especially since the AlexNet won the first prize of ILSVRC 2012 [16], deep learning has become a brilliant sub-domain.

Many convolutional neural networks (CNNs) are employed to surface defect detection, such as AlexNet [16], VGG [17], GoogLeNet [18], ResNet [19], DenseNet [20], and MobileNet [21].

The previous classification networks can only classify the defect without pointing out the region and coordinates of defect. Fast R-CNN [22] and Faster R-CNN [23] are employed to defect detection. Ref. [24] introduces a refined Fast R-CNN model and make a test on a defect dataset NEU-DET. Fast R-CNN and its variants achieve good results in practice, but they are slow.

SSD (Single Shot MultiBox Detector) [25] and YOLO (You Only Look Once) [26] are applied to handle the problem. Without requiring the region proposal stage, these methods directly generate the category probability and the position coordinate of the object, which improves the detecting speed. Ref. [27] utilizes the SSD network as the meta structure and combine it with base MobileNet for surface defect detection. Ref. [28] introduces YOLOv3 to detect multiple concrete bridge damages. Batch normalization and focal loss are incorporated in the model.

Deep learning methods have made great progress in surface defect detection. There are successful applications, especially for plane surface, such as steel plane [29], fabric plane [30], and glass plane [31]. There are also successful applications on surface defect detection in non-planar objects. Ref. [32] employes a defect detection algorithm based on a single short detector network for tiny parts in manufacturing. Ref. [33] proposes an improved YOLOv3 model named DC-TLMDDNet (Dense Connection Based Track Line Multi-target Defect Detection Network) for multi-target defect identification of the railway track line. Ref. [34] utilizes SSD and YOLO to build up a three-stage cascaded DCNN for detecting catenary support device defects. However, rare investigations have been done on copper elbow surface defect detection.

### 1.2. Objective and Structure

Copper elbows are used to link pipelines and change their direction. Copper elbows are often used for gas and oil pipeline connections. Poor quality of copper elbow can lead to severe accidents, such as natural gas leakage, fire, explosion, and so on. Therefore, the quality of copper elbow matters a great deal. It’s important to detect the defect of copper elbow accurately.

In this paper, a new defect detection model, named YOT-Net (YOLOv3 combined with triplet loss network) is proposed to deal with the copper elbow defect detection problem. The innovation of YOT-Net lies in the following aspects. Firstly, the triplet loss is introduced into the loss module of YOT-Net. To enhance feature extraction ability, the triplet loss is used as a regulation term to maximize the image similarity within the same category. Secondly, transfer learning is employed in YOT-NET, which provides a better initialization for the network. These modifications make the proposed model converge more quickly. The performance is higher than the original YOLOv3.

The remainder of this paper is organized as follows. The proposed method is discussed in detail in Section 2 . Then, experiments are presented in Section 3. Finally, Section 4 is the conclusion.

## 2. The Methodology of YOT-Net

**Figure 1:**
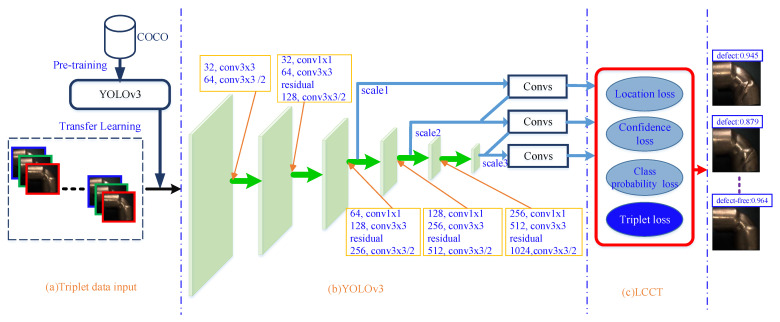
The architecture of YOT-Net. YDT-Net is a surface defect detection model and mainly composed of three basic components: (**a**) Triplet data input module. (**b**) YOLOv3 feature extract module. (**c**) LCCT (Location-Confidence-Class probability-Triplet) loss module.

Our objective was to inspect whether the copper elbow is qualified to be a product on-line. As a result, not only the detection accuracy but also the detection speed should be satisfiable. YOLOv3 is fast and accurate for target detection. Triplet loss [35] is a method to enhance feature extraction ability. A new defect detection model, named YOT-Net is proposed. YOT-Net consists of three major components, i.e., Triplet data input module, YOLOv3 module, and LCCT (Location-Confidence-Class probability-Triplet) loss module, as shown in Figure 1.

First of all, the raw images are separated into triplets. A triplet contains a base, which is either positive or negative. The input is a batch size of triplet raw images and the corresponding ground truth to the YOLOv3 network. The COCO (Microsoft Common Objects in Context) dataset [36] was used to pretrain and initialize the YOLOv3 model.

Next, image features were extracted through backbone, multiple convolutional operations, and three feature maps are outputed.

Finally, a novel loss module, named LCCT (Location-Confidence-Class probability-Triplet) loss module was constructed. The triplet loss function was applied to the loss layer as a regulation term to learn the similarity among triplet images. The loss layer was composed of location loss block, confidence loss block, class loss block, and triplet loss block. LCCT loss makes the model converge more quickly and accurately.

### 2.1. Basics of YOLOv3

**Figure 2:**
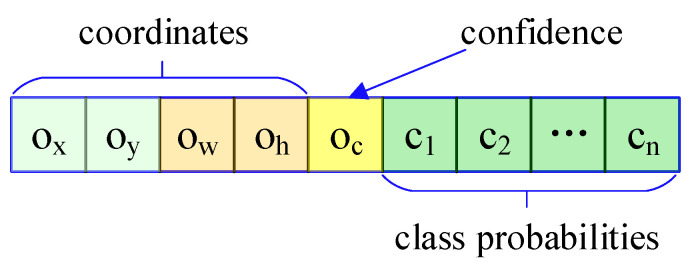
The output format of each grid.

Each input image was divided into N×N grids with the same size of the feature map. The grid in which the center of an object was located was selected to perform the prediction. Each grid predicted three bounding boxes.The output format of each grid is shown in Figure 2. The first four letters depict the location of the predicted bounding box.

**Figure 3:**
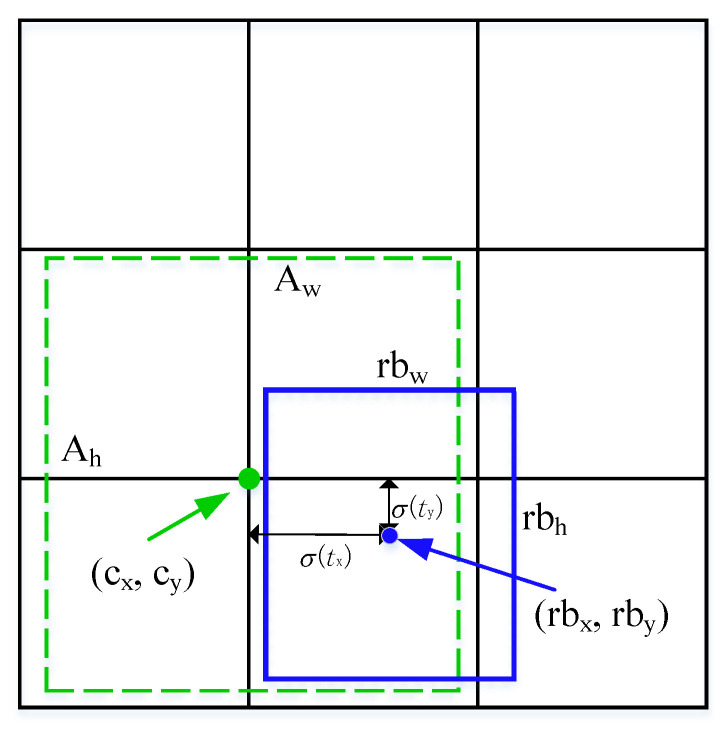
Bounding boxes for location prediction.

The cener coordinates are $\left(o_{x},o_{y}\right)$, and $o_{w},o_{h}$ are the width and height. While $o_{c}$ denotes the confidence score of the bounding box; $c_{1},c_{2},\ldots ,c_{n}$ are the probabilities of each class of object. In order to get a better prediction, prior anchor box and bounding regression were introduced to the network, as shown in Figure 3.

Assume the coordinates of the top left corner of the cell are represented by $\left(c_{x},c_{y}\right)$ and the prior anchor box has width and height $A_{w},A_{h}$, the bounding box predictions can be updated by the following formulas,

(1) $$\left\{\begin{array}{cc} rb_{x} & =\sigma \left(o_{x}\right)+c_{x}\\ rb_{y} & =\sigma \left(o_{y}\right)+c_{y}\\ rb_{w} & =A_{w}e^{o_{w}}\\ rb_{h} & =A_{h}e^{o_{h}}\\ o_{c} & =Pr\left(object\right)\times IoU_{pred}^{truth} \end{array}\right.$$

where $rb_{x},rb_{y},rb_{w},rb_{h}$ are the regression bounding box coordinates, and $\sigma \left(*\right)$ indicates the sigmoid function. Meanwhile, $o_{c}$ reflects the probability that the bounding box contains the target defect and the likelihood of the bounding box coinciding with the ground truth box (intersection-over-union, $IoU_{pred}^{truth}$). $Pr\left(*\right)$ indicates the probability of the object appearing in the box. If there is an object in the cell $Pr\left(object=1\right)$, otherwise, $Pr\left(object=0\right)$.

### 2.2. Triplet Loss

**Figure 4:**
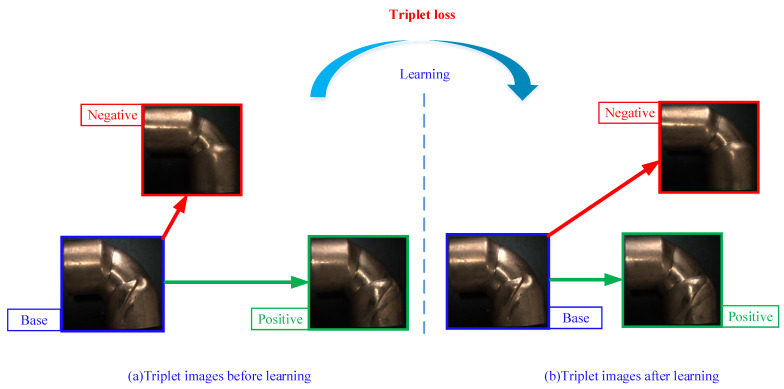
The main idea of learning with triplet loss. Triplet loss learns to minimize the distance between positive samples and the base, maximizing the distance between negative samples with the anchor. (**a**) Samples before triplet loss learning. (**b**) Samples after triplet loss learning.

Triplet loss was first proposed in [35]. A triplet data group may include three kinds of images, a base sample, a positive sample, and a negative sample. The triplet loss learns to make the distance from the base to the positive sample closer; meanwhile, making the distance to the negative sample farther. Therefore, the triplet loss was employed to improve accuracy, as shown in Figure 4.

The calculation of triplet loss is,

(2) $$loss_{triplet}=\sum_{i}^{N}\left[{∥f\left(X_{i}^{b}\right)-f\left(X_{i}^{p}\right)∥}_{2}^{2}-{∥f\left(X_{i}^{b}\right)-f\left(X_{i}^{n}\right)∥}_{2}^{2}+m\right]$$

where $f\left(*\right)$ represents the output of YOT-Net; $X_{i}^{b},X_{i}^{p},X_{i}^{n}$ stand for the base, positive sample, and negative sample; *m* refers to a margin that helps triplet loss function not be subjected to a local optimum and makes it more robust.

### 2.3. Location-Confidence-Class Probability-Triplet (LCCT) Loss Module

Triplet loss is introduced to YOT-Net as a regulation term. The YOT-Net loss function can be rewritten as Equation (3), including four parts, i.e., location loss, confidence loss, class probability loss, and triplet loss, which constitute the LCCT loss modules.

(3) $$LOSS=loss_{location}+loss_{confidence}+loss_{classprobability}+\alpha \times loss_{triplet}$$

$loss_{location},loss_{confidence},loss_{classprobability}$ are computed in a similar way as the original YOLOV3 [37]. The $loss_{triplet}$ was computed by Equation (2); α is a hyper parameter, usually set to be 0.05. By introducing the $loss_{triplet}$ to the LCCT loss module, YOT-Net can converge more quickly and predict more accurately.

Location loss is used to compute the location loss between the predict coordinates with the ground truth. It can be calculated as:

(4) $$\begin{array}{c} loss_{location}=\sum_{i=0}^{S^{2}}\sum_{j=0}^{B}I_{ij}^{obj}\left(2-w_{i}\times h_{i}\right)\left(-x_{i}\times \log\hat{x_{i}}\right)-\left(1-x_{i}\right)\times log\left(1-\hat{x_{i}}\right)\\ +\sum_{i=0}^{S^{2}}\sum_{j=0}^{B}I_{ij}^{obj}\left(2-w_{i}\times h_{i}\right)\left(-y_{i}\times \log\hat{y_{i}}\right)-\left(1-y_{i}\right)\times log\left(1-\hat{y_{i}}\right)\\ +\sum_{i=0}^{S^{2}}\sum_{j=0}^{B}I_{ij}^{obj}\left(2-w_{i}\times h_{i}\right)\left[{\left(w_{i}-\hat{w_{i}}\right)}^{2}-{\left(h_{i}-\hat{h_{i}}\right)}^{2}\right] \end{array}$$

where $I_{ij}^{obj}$ is the indicator function; $x_{i},y_{i},w_{i},h_{i}$ stand for truth bounding boxes coordinates; $S^{2}$, *B* are the number of grids in the input image and the number of bounding boxes generated by each grid, respectively. Meanwhile, $\hat{x_{i}},\hat{y_{i}},\hat{w_{i}},\hat{h_{i}}$ are the predictions. When the *i*-th cell of the *j*-th bounding box contains an object, the value is one, otherwise it is zero. Different from YOLOv2 [38], there is no hyper-parameter to increase or decrease the loss for the bounding box coordinate prediction according to the object in, or not in, the box.

The proposed model computes the confidence loss through binary cross entropy loss function as shown below:

(5) $$\begin{array}{c} loss_{confidence}=-\sum_{i=0}^{S^{2}}\sum_{j=0}^{B}I_{ij}^{obj}\left[C_{i}\log\hat{C_{i}}+\left(1-C_{i}\right)\log\left(1-\hat{C_{i}}\right)\right]\\ -\sum_{i=0}^{S^{2}}\sum_{j=0}^{B}I_{ij}^{noobj}\left[C_{i}\log\hat{C_{i}}+\left(1-C_{i}\right)\log\left(1-\hat{C_{i}}\right)\right] \end{array}$$

where $I_{ij}^{obj}$ and $I_{ij}^{noobj}$ are the indicator functions that determine whether there is an object center in the box; *C* and $\hat{C_{i}}$ stand for the ground truth confidence and the predicted confidence, respectively.

The class probability loss is calculated in the same way of confidence loss.

(6) $$loss_{classprobability}=\sum_{i=0}^{S^{2}}\sum_{j=0}^{B}I_{ij}^{obj}\left(\sum_{c\in classes}\left[p_{i}\left(c\right)\;log\hat{p_{i}}\left(c\right)+\left(1-p_{i}\left(c\right)\right)\;log\left(1-\hat{p_{i}}\left(c\right)\right)\right]\right)$$

where *c* is the class; $p_{i}\left(c\right)$ and $\hat{p_{i}}\left(c\right)$ stand for the truth class probability and predicted class probability in *i*-th box, respectively.

## 3. Experiments and Analysis

### 3.1. Dataset

First of all, the manufacturers hope to pick out the defect elbows. Furthermore, they also would like to know what is the distribution of each kind of defect, so that the reason causing the defect with the maximum proportion could be analyzed in time.

In order to perform these two tasks, two new surface defect detection datasets, named “TJ-CE-CLS” and “TJ-CE-DET”, are construed in this paper. The TJ-CE-CLS dataset includes 317 images, i.e., 229 samples in the defective class and 88 samples in the defect-free class. The TJ-CE-DET dataset includes 229 images containing of 384 defects, i.e., 131 samples in the extrusion class, 85 samples in the crack class, and 168 samples in the pitted-surface class.

Copper elbow defect images were collected from a copper elbow manufacturer.

**Figure 5:**
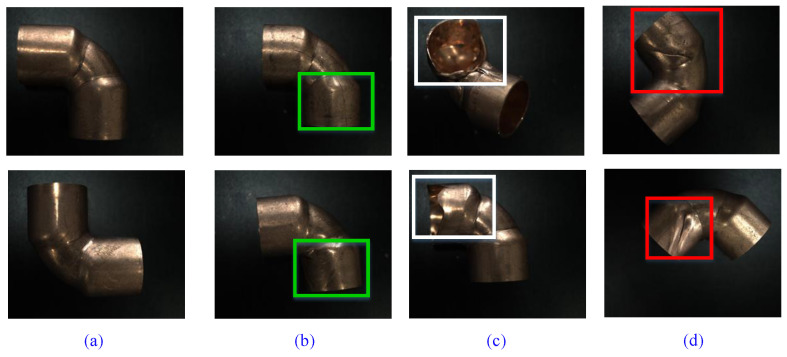
Some images of the copper elbow surface. (**a**) Defect-free. (**b**) Extrusion. (**c**) Crack. (**d**) Pitted-surface.

Some samples of copper elbow are shown in Figure 5. The defect images are RGB images, with a scale of 2456 × 2048. There are three major defects on the copper elbow surface, i.e., extrusion, crack, and pitted-surface.

### 3.2. A View of Results

YOT-Net was implemented on the open source deep learning toolbox TensorFlow [39]. The model runs on a NVIDIA GTX 1080TI GPU (with 11GB memory) with Ubuntu 18.04 Linux.

Transfer learning was introduced in the training pipeline. The proposed model was pretrained on the COCO dataset and then trained on the TJ-CE-CLS and TJ-CE-DET datasets. YOT-Net was trained with a learning rate of 0.00001, weight decay of 0.9995, and momentum of 0.9. The exponential moving average was applied to the training process, which makes the model more robust. The leaky ReLU function was selected as the activation function.

**Figure 6:**
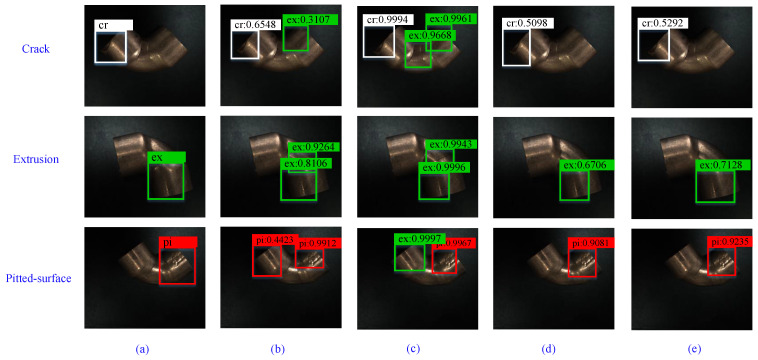
A brief view of the prediction results on TJ-CE-DET. There is only one defect in each image. SSD and Faster RCNN predict two or three defects though they get higher scores. YOT-Net gets higher scores than the Original YOLOv3. (**a**) Ground truth, (**b**) SSD, (**c**) Faster R-CNN, (**d**) YOLOv3, (**e**) our proposed method.

For comparison, SSD, Faster R-CNN, YOLOv3, and YOT-Net were carried out on the same dataset, as shown in Figure 6, and the detection results are given in columns 2 to 5. The first column are the ground truth defects. It’s clear that SSD and Faster R-CNN got higher scores than YOLOv3 and YOT-Net, but they are more likely to make mistakes. Take the first image of the third column as an example. There is only one crack defect on the image, but Faster R-CNN predicts three defects, one crack defect, and two extrusion defects. This mistake will lead to a high false negative rate, and make more normal copper elbows to be classified into the defect group. YOT-Net shows more robustness.

### 3.3. Performance Evaluation

#### 3.3.1. Evaluation Metrics

Accuracy, precision, recall, F1 score, false positive rate (FPR), false negative rate (FNR), and mean average precision (mAP) were employed to evaluate the performance of YOT-Net.

#### 3.3.2. Comparisons with the Other Classification Methods

Experiments were conducted on the TJ-CE-CLS dataset to compare the proposed model with some popular classification methods, including Bayesian [9], KNN [10], SVM [12], AlexNet [16], DenseNet [20], VGG [17], ResNeXt [40], as shown in Table 1 and Table 2.

Table 1 shows that YOT-Net outperforms the traditional machine learning methods. The defects of copper elbow in the dataset are quite challenging. For example, extrusion in the defect image is hard to classify for traditional methods and are sensitive to the illumination in the image. In addition, traditional methods feature extraction abilities that are inferior to deep learning methods, especially in the domain of computer vision.

YOT-Net gets better classification, while requiring more computational resources and spending much more time on inferences, as shown in Table 1. This better performance benefits from the outstanding feature extracting ability of the deep learning method. Therefore, another group of experiments are carried out to compare YOT-Net with other deep learning classification methods.

Experiment results show that the proposed YOT-Net also achieves the best results in accuracy, precision, and F1 score. Firstly, the YOT-Net model is bigger than AlexNet, DenseNet, and ResNeXt, so it possesses more representation ability. The second reason is that the LCCT loss module in the YOT-Net forces the model to extract more useful features. The triplet loss function forces YOT-net to learn to classify the same category image of triplet samples. After that, YOT-Net behaves with the ability to maximize the distance of different categories, which is helpful in the last inference procedure.

Under the concept of FNR and FPR, the lower rate means a better result. The FPR, despite YOT-Net, is 7.41% higher than DenseNet and VGG, the performance of YOT-Net is also acceptable. For FNR, YOT-Net gets at least a 7.4% lower prediction rate than others. FNR is crucial to the copper elbow, because a non-defective group of products do not go for another check. That means a copper elbow with defects will be sent out to the customer if it is mixed in this group.

Otherwise, YOT-Net takes more time to detect an image, because YOT-Net not only gives the probabilities but also the coordinates.

#### 3.3.3. Comparisons with Deep Learning Detection Methods

Experiments were conducted to compare the proposed model with Faster R-CNN [23], SSD [25], and YOLOv3 [37], as shown in Table 3. Faster R-CNN and SSD were implemented with the mmdetection toolbox [41].

YOT-Net got the highest mAP among those methods involved in comparison. With regard to other indicators, YOT-Net also behaves satisfactory. Although FNR of YOT-Net is a little higher than that of Faster R-CNN, YOT-Net saves about 42.65% time. Fast inference is helpful for elbow defect detection online.

#### 3.3.4. Proportion of Each Kind of Defect

In the TJ-CE-DET dataset, defects are specified to be three types, e.g., extrusion, crack, and pitted-surface.

Under YOT-Net, the proportion of each kind of defect to the total detects is easily known, as shown in Table 4. This number if helpful for checking the reason causing the defects.

### 3.4. Ablate Study

#### 3.4.1. The Convergence Speed of YOLOv3 and YOT-Net

An experiment with 50 epochs, 0.00001 learning rate, and 0.9995 decay rate was carried out to study the convergence speed of YOLOv3 and YOT-Net.

**Figure 7:**
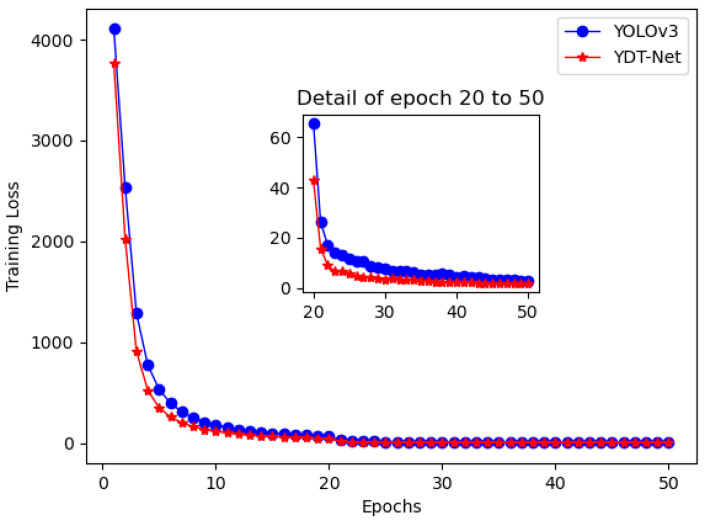
The convergence speed of YOLOv3 and YOT-Net. The red line, refers to YOT-Net, and converges more quickly than blue line for YOLOv3.

As shown in Figure 7, YOT-Net converges more quickly than the original YOLOv3. The reason is that the triplet loss help the YOT-Net learn similarity and dissimilarity of triplet data within an epoch. This accelerates the convergence progress.

#### 3.4.2. The Margin of Triplet Loss

**Figure 8:**
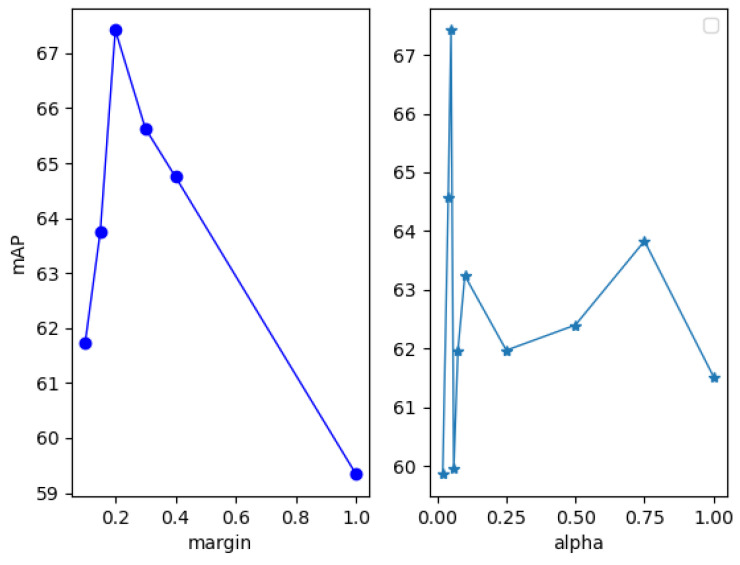
The effect on mAP of different values in margin and alpha. The **left** plot shows the effect of margin on mAP. The **right** plot shows the effect of alpha on mAP.

In order to get a small triplet loss, the distance between the base and the positive sample is minimized; meanwhile, the distance between the base and negative sample is maximized. The hyper parameter *m* is a key factor to the loss function [35]. In this paper, a series of experiments were conducted to find an appreciate value, and the experiment results show that 0.2 is a good choice, as shown in Figure 8.

#### 3.4.3. The Hyper Parameter α in LCCT Loss Module

As shown in Equation (3), loss_triplet is a regular term to enhance feature extract ability. It’s also key to set the hyper parameter α in Equation (3). A series of experiments were set up to get a well-performed value. As shown in Figure 8, 0.05 is a satisfactory value for the hyper parameter α.

## 4. Conclusions

The copper elbow surface defect detection is an important and challenging task. In this paper, a YOLOv3 combined triplet loss network(YOT-Net) is proposed to tackle this problem. The introduction of triplet loss greatly improves the feature extracting ability. Experiments were carried out on practical copper elbows. On distinguishing defect elbows from non-defect ones regardless of the type of defects, YOT-Net performs quite well on accuracy, precision, recall rate, and F1 score. Meanwhile, the proportion of each kind of defect to the total defect can be easily known. This is helpful for checking the reason causing different kinds of defects. In addition, YOT-Net shows faster convergence rates.

## Figures and Tables

**Table 1:** Comparisons with the traditional classification methods.

Indicator	Naive Bayes	KNN	SVM	YOT-Net
Accuracy	51.56	62.5	73.44	84.38
Precision	27.59	31.25	53.85	73.33
Recall	44.44	27.78	38.89	64.71
F1 score	34.04	29.41	45.16	68.75
FPR	55.56	72.22	61.11	35.29
FNR	45.65	23.91	13.04	8.51
Inference time (ms per image)	0.3	33.9	44.5	215.7

**Table 2:** Comparisons with the deep learning classification methods.

Indicator	AlexNet	DenseNet	VGG	ResNeXt	YOT-Net
Accuracy	67.74	69.35	74.19	74.19	84.38
Precision	45.45	48.15	54.17	56.25	73.33
Recall	55.56	72.22	72.22	50.00	64.71
F1 score	50.00	57.78	61.9	52.94	68.75
FPR	44.44	27.78	27.78	50.00	35.29
FNR	27.27	31.82	25.00	15.91	8.51
Inference time (ms per image)	56.9	68.2	62.9	80	215.7

**Table 3:** Comparisons with other detection methods.

Indicator	Faster R-CNN	SSD	YOLOv3	YOT-Net
Accuracy	82.81	75	79.69	84.38
Precision	75	53.33	64.29	73.33
Recall	52.94	47.06	52.94	64.71
F1 score	62.07	50	58.06	68.75
FPR	47.06	52.94	47.06	35.29
FNR	6.38	14.89	10.64	8.51
mAP	57.89	60.41	59.19	67.42
Inference time(ms per image)	307.7	250	215.7	215.7

**Table 4:** The ratio of extrusion, crack, and pitted-surface in TJ-CE-DET.

Extrusion	Crack	Pitted-Surface
22.13%	34.11%	43.75%

## Data Availability

Not applicable.
